# *Pseudomonas aeruginosa* Keratitis in Rats: Study of the Effect of Topical 5% Hesperidin Practice on Healing

**DOI:** 10.5152/eurasianjmed.2023.22234

**Published:** 2023-02-01

**Authors:** Bahadır Utlu, Osman Öndaş, Mustafa Yıldırım, Kemal Bayrakçeken, Serkan Yıldırım

**Affiliations:** 1 Department of Ophthalmology, Erzurum Regional Training and Research Hospital, Erzurum, Turkey; 2 Department of Ophthalmology, Atatürk University School of Medicine, Erzurum, Turkey; 3 Department of Ophthalmology, Binali Yıldırım University School of Medicine, Erzincan, Turkey; 4 Department of Pathology, Atatürk University Faculty of Veterinary, Erzurum, Turkey

**Keywords:** Boric acid, Pseudomonas aeruginosa, keratitis, inflammation

## Abstract

**Objective:**

The aim of this study is to investigate the effect of topical 5% hesperidin application on healing.

**Materials and Methods:**

After 48 rats were randomized and divided into 7 groups, on the first day, an epithelial defect was created in the center of the cornea with the help of microkeratome under intraperitoneal ketamine + xylazine and topical 5% proparacaine anesthesia for the groups to be infected with keratitis according to the groups mentioned below. An amount of 0.05 mL of the solution containing 10^8^ colony-forming units/mL *Pseudomonas aeruginosa* (PA-ATC27853) will be taken and inoculated per rat. At the end of the 3 days incubation period, rats with keratitis will be added to the groups, and active substances and antibiotics will be given topically together with other groups for 10 days. At the end of the study, the ocular tissues of the rats will be removed and examined histopathologically.

**Results:**

A clinically significant reduction in inflammation was detected in the groups using hesperidin. No transforming growth factor-β1 staining was detected in the group in which keratitis + hesperidin was treated topically. In the group in which hesperidin toxicity was examined, mild inflammation and thickening of the corneal stroma layer were observed, and it was evaluated as a negative transforming growth factor-β1 expression in the lacrimal gland tissue. Corneal epithelial damage was minimal in the keratitis group, and the toxicity group was treated with only hesperidin when compared to the other groups.

**Conclusion:**

Topical hesperidin drops may be an important therapeutic factor in tissue healing and in the fight against inflammation in the treatment of keratitis.

**Main Points**

- Hesperidin can be considered a different treatment modality in patients with severe keratitis.
- Hesperidin has been shown to have an important potential in terms of antibacterial and anti-inflammatory activity in *Pseudomonas aeruginosa* keratitis.
- It has been shown that no toxic effect was observed, in which normal corneal tissue exposed to hesperidin remained largely intact.
- With the use of hesperidin-like molecules in topical treatment, complications that will adversely affect the clinical prognosis can be prevented.

## Instruction

Flavonoids are polyphenolic compounds synthesized by plants and have many pharmacological activities. They act as powerful free radical scavengers that protect the human body, and they do this through the OH group in its molecular structure. It contains 6 main classes: flavonols, flavones, flavanones, catechins, anthocyanidins, and isoflavones.^1^ Hesperidin is a natural cost-effective flavanone glycoside, which is found in large quantities in citrus fruits such as oranges and lemons. Hesperidin plays an important role in the prevention of many diseases due to its anti-inflammatory, anti-oxidant, anti-ulcer, and anti-carcinogenic activities,^2,3^ in addition to its anti-melanogenic and neuroprotective effects.^4,5^ Moreover, it has anti-microbial, anti-allergic, immunomodulatory, hepatoprotective, and nephroprotective effects.^6-8^ The curative role of hesperidin against tissue toxicities caused by various chemotherapeutic agents such as doxorubicin, methotrexate, and cisplatin has been reported.^9-11^ The therapeutic role of hesperidin in addition to its anti-cataractogenic effects in various ocular disorders such as diabetic retinopathy and diabetic macular edema was demonstrated in previous studies.^12^

*Pseudomonas aeruginosa* is of particular concern among the organisms that cause bacterial keratitis for a variety of reasons. *P. aeruginosa* is responsible for 6%-39% of bacterial keratitis cases in the United States and 8%-21% in Southern India.^13^ In addition, *P. aeruginosa* corneal ulcers are observed more severe than other bacterial corneal ulcers.^14^ Furthermore, *Pseudomonas* causes corneal thinning at a level that will lead to serious abscesses and perforations in the cornea with the toxins it secretes. *P. aeruginosa* is highly lethal, and *P. aeruginosa* ulcers and abscesses are often more difficult to treat and are known to cause worse visual outcomes than other bacterial corneal ulcers.

It was aimed in our study to evaluate the potential protective role of hesperidin against ocular effects caused by *Pseudomonas *keratitis in rats using different histological and immunohistochemical techniques. Moreover, it was aimed to compare hesperidin treatment with classical treatment methods and to examine whether hesperidin could have a toxic effect on the cornea.

## Materials and Methods

The study was approved by Atatürk University Veterinary Animal Experiments Local Ethics Committee (Approval no: 75296309-050.01.04-E.1800377638-234). The study adheres to the ARVO Statement for the Use of Animals in Ophthalmic and Vision Research. The study was started with 48 female Sprague-Dawley rats (200-400 g). The rats were randomized into 6 groups.

**Figure 1.**
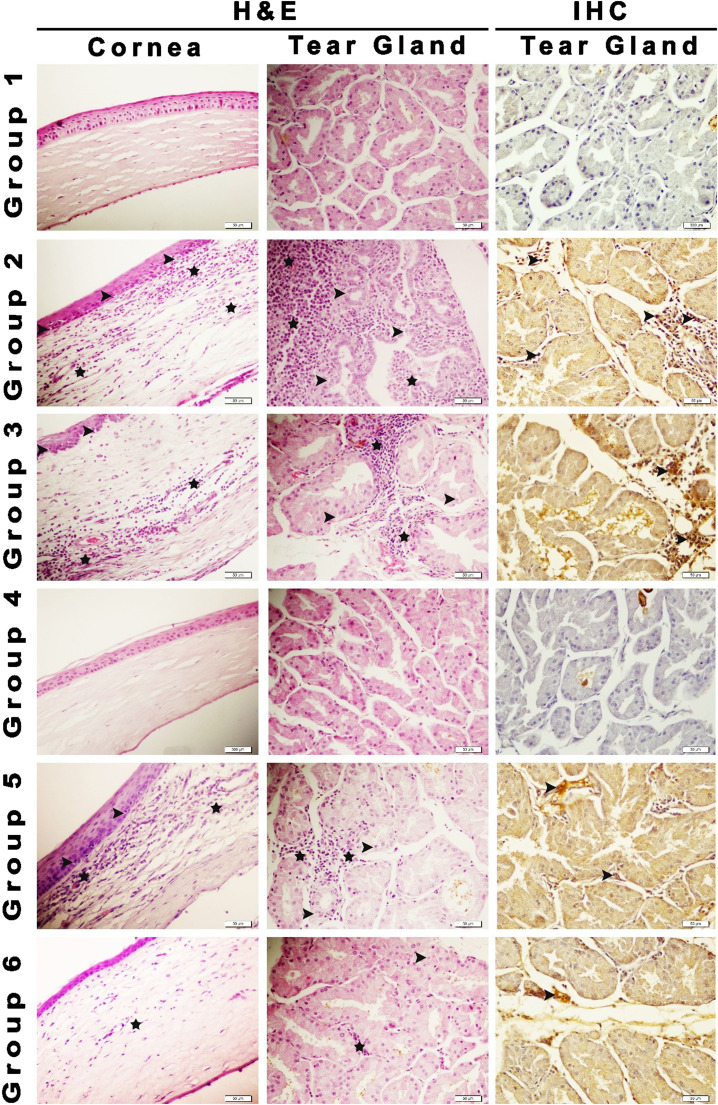
Cornea and lacrimal gland tissues, degeneration of corneal epithelium (arrowheads), inflammation of stroma (stars), degeneration of glandular epithelium (arrowheads), inflammation of interstitial spaces (asterisks), H&E, cytoplasmic TGF-β1 expressions in inflammatory cells around vessels and interstitial spaces (arrowheads), IHC-P (Immunohistochemistry- Parafin Protocol), Bar: 50 μm. H&E, hematoxylin and eosin; TGF-β1, transforming growth factor-β1.

- Group 1 (control group = C): Eight rats were determined as the control group.
- Group 2 (*Pseudomonas *keratitis(+)): A corneal epithelial defect was created in 8 rats under anesthesia on the first day and 0.05 mL per rat was inoculated from a solution containing 1 × 10^8^ colony-forming units (CFU)/mL *P*. *aeruginosa* (PA-ATC 27853), and those with keratitis at the end of the third day were added to the group. No topical treatment was practiced for 10 days (Figure 1).
- Group 3 (***Pseudomonas ***keratitis(+)+vancomycin + ceftazidime drop therapy 5 × 1): On the first day, a corneal epithelial defect was created in 8 rats under anesthesia and 0.05 mL per rat was inoculated from a solution containing 1 × 10^8^ CFU/mL *P*. *aeruginosa* (PA-ATC 27853), and those with keratitis at the end of the third day were added to the group. In this group, *vancomycin+ceftazidime drops were administered topically for 10 days, 5× 1* separately.
- Group 4 (***Pseudomonas ***keratitis(+) + 5% hesperidin drop therapy 2× 1): *On the first day, a corneal epithelial defect was created in* 8 rats under anesthesia, and 0.05 mL per rat was inoculated from a solution containing 1 × 10^8^ CFU/mL *P*. *aeruginosa* (PA-ATC 27853); at the end of the third day, the rats with keratitis were added to the group.
  - In this group, 8% boric acid was administered topically for 10 days as 2× 1 drops.
- Group 5 (***Pseudomonas ***keratitis(+) + vancomycin drop 5× 1 + ceftazidime drop therapy 5× 1 + 5% hesperidin drop therapy 2× 1 (n = 8)): On the first day, a corneal epithelial defect was created in 8 rats under anesthesia, and 0.05 mL per rat was inoculated from a solution containing 1 × 10^8^ CFU/mL *P*. *aeruginosa* (PA-ATC 27853); at the end of the third day, the rats with keratitis were added to the group.
  - In this group, vancomycin + ceftazidime drops 5× 1 + 5% hesperidin drops were applied topically for 10 days as 2× 1 drops separately.
- Group 6 (5% hesperidin drop therapy 2× 1 (toxicity group) (n = 8)): 5% hesperidin was administered topically as 2× 1 drops to 8 rats.
  - On the 14th day, which was the end of the study, the rats were sacrificed, their globule structures were removed as a whole and stored in 10% formaldehyde, and the sacrificed rats were examined microscopically by hematoxylin-eosin and immunohistochemical staining by a pathologist who was unaware of the groups, and the results were evaluated.

### Histopathological Examination

Tissues taken as a result of the study were fixed in 10% formalin solution for 48 hours. Then, after routine tissue follow-up procedures, paraffin blocks were prepared. Preparations were prepared by taking incisions of 4 mm thickness from each block. In order to examine their general appearance, the preparations were stained with hematoxylin-eosin and examined under a light microscope. According to the lesions, the preparations were examined under the light microscope; they were evaluated as none (−), mild (+), moderate (++), and severe (+++) and photographed.

All sections taken on slides with adhesive (poly-l-lysine) for immunoperoxidase examination were passed through xylol and alcohol series. The sections were washed with phosphate-buffered saline (PBS) and incubated in 3% H_2_O_2_ for 10 min, thereby inactivating endogenous peroxidasin. In order to reveal the antigen in the tissues, they were treated with antigen retrieval solution for 2× 5 minutes at 500 W in a microwave oven and allowed to cool. Tissues washed with PBS were then incubated at 37°C for 30 minutes with transforming growth factor-β1 (TGF-β1) antibody (Catalog no: sc-130348, dilution 1/50; Santa Cruz, USA) for the detection of DNA damage. They were incubated with biotinized secondary antibodies for 10 minutes at room temperature. Incisions washed again with PBS were incubated in streptavidin-peroxidase for 10 minutes, and then, they were washed with PBS in the same way. 3,3’-Diaminobenzidine (DAB) was used as a chromogen. It was washed with distilled water. Counterstaining was applied with hematoxylin (Mayer’s) for 15-20 seconds. It was passed through the xylol and alcohol series and covered with a coverslip with the help of Entellan. The incisions were evaluated according to their immune positivity as in the histopathological examination.

### Preparation of Topical Agents

β-Cyclodextrin (β-CD) was used to increase the solubility of hesperidin. Hesperidin solution was prepared by dissolving sodium hydroxide and adding this solution to 10% β-CD (prepared in IPBS (Isotonic phosphate-buffered solution), pH 7.4, containing 0.1% hydroxypropyl methylcellulose). The pH was fixed to 7.4 using hydrochloric acid.^15^ Vancomycin + ceftazidime fortification drops were prepared by diluting 1 g vancomycin and ceftazidime in 10 cc vials at a dilution ratio of 2/5.

### Statistical Analysis

All statistical analyses were performed using Statistical Package for Social Sciences (SPSS) for Windows, v. 18.0 software package (IBM SPSS Corp.; Armonk, NY, USA), and a *P*-value of <.05 was accepted as statistically significant. Histopathological findings were analyzed with SPSS v. 20.00. The differences between the groups were determined using the Kruskal–Wallis test, a non-parametric method, and the Mann–Whitney *U*-test was conducted to identify the group that created the significant difference (*P* < .05).

## Results

### Histopathologic Findings

**Figure 2.**
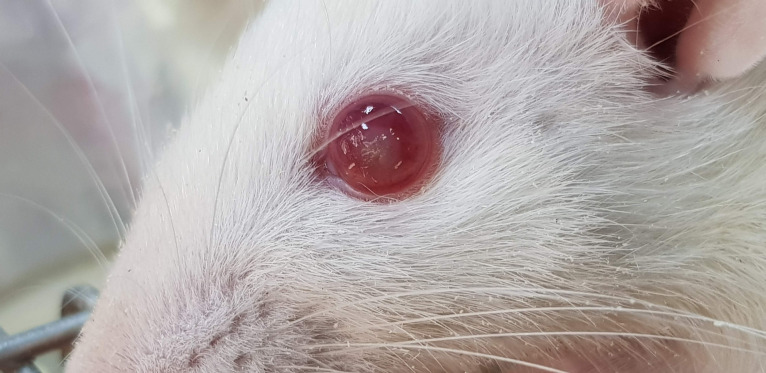
The rats that started keratitis at the end of the third day were included in the study.

Group 1 (control group = C): The examined corneal and lacrimal gland tissues were found to have a normal histological appearance (Figure 2).

Group 2 (***Pseudomonas ***keratitis(+)): In the examined cornea and lacrimal gland tissues, severe corneal thickening, severe mononuclear cell infiltrations in the stroma layer, degeneration, and necrosis in the corneal epithelium were determined. Degeneration of the lacrimal gland epithelium and severe inflammation were observed in the interstitial spaces and gland lumens (Figure 2).

Group 3 (***Pseudomonas*** keratitis(+)+vancomycin+ceftazidime drop therapy 5× 1): Histopathological examination of the examined corneal and lacrimal gland tissues revealed moderate thickening of the corneal wall and moderate degeneration of the corneal epithelium (Figure 2). Moderate degeneration of the tear gland epithelium and moderate inflammation in the interstitial spaces were observed (Figure 2).

Group 4 (***Pseudomonas*** keratitis(+) + 5% hesperidin drop therapy 2× 1): The examined corneal and lacrimal gland tissues were found to have a normal histological appearance (Figure 2).

Group 5 (***Pseudomonas*** keratitis(+) + vancomycin drop 5× 1 + ceftazidime drop therapy 5× 1 + 5% hesperidin drop therapy 2× 1 (n = 8)): When the cornea and lacrimal gland tissues were examined histopathologically, moderate thickening of the cornea and moderate inflammation in the stroma layer were observed. Moderate degeneration was detected in the lacrimal glands and moderate inflammation in the interstitial spaces (Figure 2).

Group 6 (5% hesperidin drop therapy 2× 1 (toxicity group) (n = 8)): When the cornea and lacrimal gland tissues were examined histopathologically, mild inflammation and thickening were observed in the stroma layer of the cornea. *Mild degeneration *and interstitial inflammation were observed in the gland epithelium (Figure 1). The histopathological findings are summarized in Table 1.

### Immunohistochemical Findings

Group 1 (control group = C): When the lacrimal gland tissues were examined immunohistochemically, negative TGF-β1 expression was observed (Figure 1).

Group 2 (***Pseudomonas***** keratitis(+)): **When the lacrimal gland tissues were examined immunohistochemically, severe TGF-β1 expression was observed in the cytoplasm of inflammatory cells in the interstitial spaces and around the vessels (Figure 2).

Group 3 (***Pseudomonas*** keratitis(+) + vancomycin + ceftazidime drop therapy 5× 1 ): When the lacrimal gland tissues were examined immunohistochemically, moderate TGF-β1 expression was determined in the interstitial spaces, in the inflammatory cell cytoplasm, and around the vessels (Figure 2).

Group 4 (***Pseudomonas*** keratitis(+)+5% hesperidin drop therapy 2× 1): When the lacrimal gland tissues were examined immunohistochemically, negative TGF-β1 expression was determined (Figure 2).

Group 5 (***Pseudomonas*** keratitis(+) + vancomycin drop 5× 1 + ceftazidime drop therapy 5× 1 + 5% hesperidin drop therapy 2× 1 (n = 8)): When the lacrimal gland tissues were examined immunohistochemically, moderate TGF-β1 expression was observed in the interstitial spaces, in the inflammatory cell cytoplasm and around the vessels (Figure 2).

Group 6 (5% hesperidin drop therapy 2× 1 (toxicity group) (n = 8)): When the lacrimal gland tissues were examined immunohistochemically, mild TGF-β1 expression was detected in inflammatory cells in the interstitial spaces (Figure 2). A statistically significant difference (*P* < 0.05) was found when compared with the infected group (Table 1).

## Discussion

Bacterial keratitis is common in the cornea tissue and is sight-threatening. Ineffective or delayed treatment of this disease, which is common among infectious eye diseases, may first cause endophthalmitis and then eye loss.^15,16^ A large number of microorganisms cause bacterial keratitis. While the most common pathogen isolated from bacterial corneal ulcers was *Streptococcus pneumonia* previously, the frequency of *Pseudomonas* and *Staphylococcus* infections has increased with the increase in contact lens use.

Systemic and/or local ocular antibiotic administration is the routine treatment for bacterial keratitis. However, while the blood–ocular barrier prioritizes local treatment instead of systemic treatment, the number of antibiotics to be used is limited due to the low corneal penetration of local drugs. In addition, one of the factors that complicates the treatment of bacterial keratitis is the increase in microbial resistance to a small number of antibiotics.^17,18^ Therefore, the search for more effective natural therapeutic agents to treat ocular bacterial infections is inevitable.

New antibiotics/antiseptics, the discovery of new ocular agents, can be obtained through chemical synthesis or discovery, which will basically be achieved with the use of plant extracts. Today, the decrease in the number of synthetic molecules has increased the interest of researchers in naturally derived molecules. Hesperidin is a type of flavonoid with a wide variety of pharmacological properties and effects. It also has powerful antioxidant, anti-inflammatory, antibacterial, and free radical scavenging activities,^19, 20, 21, 22^ according to current research reports. Studies have shown that hesperidin reduces inflammatory mediators and exerts significant antioxidant effects. They stated that the molecular basis of its anti-inflammatory effects is mediated by specific signaling pathways, including the nuclear factor κβ pathway.^23^ In our study, local application of hesperidin was shown to be effective in the treatment of cornea and lacrimal gland infections of rats with *Pseudomonas* keratitis. Although mild inflammatory findings occurred in the toxicity group, no diffuse inflammation was detected in the ocular tissues.

Hesperidin is a bioflavonoid and has poor water solubility due to its hydrophobic nature, which creates challenges in topical ocular application. However, it has been reported that hesperidin solubility in liquids and penetration into ocular tissues are provided by the use of cyclodextrin molecules and targeted liposomes as a drug delivery system.^24^ A pharmacokinetic study in humans has shown that HP (Hesperidin) migrates to ocular tissues (choroid, sclera).^25^ The disadvantage of this situation is the use of these formulations, their low storage stability in solution, their complexity, and their high preparation costs.^25,26^ It has been confirmed that many plant components have strong antibacterial activities as well as strong anti-inflammatory and antioxidant activities. In addition, many studies have confirmed that anti-inflammatory and antioxidant activities may further contribute to the therapeutic effects of antibiotics.^27^ Antibiotics of natural origin are also seen to be safe and largely free of side effects.^28^

In recent studies, it has been shown that hesperidin has antimicrobial activity, and the protection of hesperidin against toxicities caused by some pathogens and cytotoxic agents has been evaluated. The mechanisms of its antimicrobial properties are not clear as day. However, various mechanisms have been proposed, such as penetration of bacterial walls, inhibition of microbial enzymes, and activation of the immune system. Furthermore, the lipophilicity of flavonoids is thought to be an important factor for antimicrobial activity.^29^

In another study, it was found that hesperidin-loaded biopolymer particles showed antibacterial effects against *Escherichia coli*, *Klebsiella pneumoniae*, and *P. aeruginosa*, and their antimicrobial effects increased with increasing concentration. The antimicrobial susceptibility results showed that hesperidin-loaded biopolymer particles have a broad-spectrum antibiotic potential in the inhibition of both Gram-positive and Gram-negative bacteria.^30^

In a study investigating the anti-hepatic effect of hesperidin, it was shown that hesperidin could significantly improve the TGF-β1 response. In another study, in which they aimed to evaluate the synergistic role of hesperidin supplementation and fasting regimen on aging and inflammatory biomarkers, it was discovered that hesperidin supplementation significantly depressed inflammatory cytokines.^31^ In our study, we demonstrated the inflammatory effect of hesperidin on another ocular surface tissue, the lacrimal gland, in accordance with the literature. Although the results are in accordance with the literature, TGF-β1 immunohistochemical staining could not be demonstrated due to the avascular structure and fragile tissue of the cornea. This is seen as the missing aspect of our study.

In conclusion, bacterial keratitis is a condition that becomes increasingly difficult to treat and causes severe ocular morbidity. Treatment becomes more difficult due to increasing antibiotic resistance. Topical administration of hesperidin, which has anti-inflammatory and antibacterial effects, can be a part of local treatment in combating bacterial keratitis.

## Figures and Tables

**Table 1.** Scoring of Histopathological and Immunohistochemical Findings in Eye and Tear Gland Tissues

	Group 1	Group 2	Group 3	Group 4	Group 5	Group 6
Thickening of the cornea	**−**	**+++**	**++**	**−**	**++**	**+**
Corneal epithelial damage	**−**	**+++**	**++**	**−**	**+**	**−**
Corneal inflammation	**−**	**+++**	**++**	**−**	**++**	**+**
Gland epithelial damage	**−**	**+++**	**++**	**−**	**+**	**−**
Gland inflammation	**−**	**+++**	**++**	**−**	**++**	**+**
TGF-β1	**−**	**+++**	**++**	**−**	**++**	**+**

TGF-β1, transforming growth factor-β1.
